# Social-Emotional Difficulties in Irish Children Aged Five and Nine Years: A National, Longitudinal Study

**DOI:** 10.3390/children8080656

**Published:** 2021-07-29

**Authors:** Ann Swift, Roy McConkey, Philip Curry, Edurne Garcia Iriarte

**Affiliations:** 1 School of Social Work and Social Policy, Trinity College Dublin, Dublin 2, Ireland; aswift@tcd.ie (A.S.); pcurry@tcd.ie (P.C.); iriartee@tcd.ie (E.G.I.); 2 Institute of Nursing and Health Research, Ulster University, Newtownabbey BT37 0QB, UK

**Keywords:** social-emotional difficulties, children, Ireland, developmental impairments, family caregivers, strength and difficulties questionnaire, growing up in Ireland

## Abstract

A small proportion of children experience social-emotional difficulties from early childhood onwards. Longitudinal studies with nationally representative samples are needed to identify the prevalence and the characteristics of children and families persistently experiencing these difficulties. Secondary analysis of data collected on over 7500 Irish children and with the Strengths and Difficulties Questionnaire as the primary indicator, found that 6% of children when they were five year olds and 8% when they were nine-years, had above threshold scores that warranted further investigation. A smaller proportion—2.9% had elevated scores at both ages. Logistic regression analyses found that children with one or more developmental disabilities were up to six times more likely to have sustained difficulties. There were also significant associations with the lower education attainment of primary caregivers and the socio-economic deprivation of families. Primary caregivers and teachers reported higher conflict in their relationships with these children. Although the number of Irish children presenting with continuing social-emotional difficulties is small, they can present an ongoing and future societal cost in terms of the impact on family relations and demands placed on educational, health and social services. This study identified the children and families who are at greatest risk and for whom targeted early intervention services could be provided.

## 1. Introduction

Social-emotional difficulties are experienced throughout childhood and into adolescence. For most children they are transient, but a minority will experience more severe and sustained difficulties, with the numbers increasing with age [1]. These can take various forms, including emotional disorders such as anxiety and depression, conduct disorders including anti-social behaviors, hyperactivity and inattention, and poor peer-relationships [2]. Such difficulties can impact greatly on children’s lives. They are associated with poorer health and greater use of health and social services, low educational achievements, poor employment prospects, with increased risk of delinquency and substance abuse [3].

Social-emotional problems occur more frequently with children who have developmental impairments such as physical and intellectual disabilities and autism spectrum disorders [4]. These are apparent from three years onwards, especially with boys [5]. But these impairments have to be considered alongside the social and environmental influences on children’s development as identified in the International Classification of Functioning for children and young people (ICF-CY) [6]. This includes social factors within the family such as income levels and parental education as well as the emotional wellbeing of parents and parent-child interactions that can be a response to, as well as a contributor to child’s social-emotional difficulties [7].

Early identification of socio-emotional difficulties should facilitate earlier intervention strategies although to date these have been mainly limited to child-centred, behavioral therapies [8]. However, there is a growing awareness of the need for family-based interventions to commence at an earlier age in order to ameliorate children’s behaviour becoming more chronic and harder for families and professionals to address [9].

Research to date has been focused mainly on adolescents. Yet a number of recent studies have shown that these difficulties can be manifest in preschool and early childhood years. A cross sectional study of kindergarten children in Canada reported up to 40% had ‘socio-emotional vulnerabilities’ with 9% having multiple vulnerabilities, mostly higher aggression and hyperactivity [10]. In a Scottish longitudinal study involving 2500 parents of four-year olds, 30% were reported to have behavioural difficulties—more commonly in boys—and when the children were six years of age, 18% continued to have such difficulties. The likelihood of children having these difficulties was greater with single parent status, boys and higher level of socio-economic deprivation [11]. A Danish study of over 1000 children aged between 5 and 7 years rated by parents on the Strengths and Difficulties Questionnaire (see below) identified around 10% as having probable diagnostic significant scores. When the children were formally assessed at age 12, they had a significantly higher odds of being identified with hyperkinetic and inattentive disorders but less for emotional disorders [12].

One of the few studies undertaken with nationally representative samples of children took place in Ireland with a cohort of over 7500 children at age 9 and again at age 13 [13]. Parental ratings on the Strengths and Difficulties questionnaire identified 7% of nine-year olds as having above the threshold scores indicative of probable diagnostic significance which had declined slightly to 6.3% at age 13. However the majority of children (*n* = 6598 had below threshold scores at both ages (89.5%) with 558 children (7.6%) experiencing a change in SDQ outcomes (whether an improvement or dis-improvement) between the two ages. The remaining 2.9% (216 children) had above threshold scores at both ages.

The data from a second national cohort of Irish children at ages 5 and 9 nine years has become available with similar measures relating to children’s social-emotional difficulties based on parental reports as well as teacher ratings. This provided an opportunity to extend to a younger age, the previous analyses with children aged 9 and 13 years and to replicate the findings across two cohorts and with two raters of children’s difficulties. This would add to the robustness of the findings and make a unique contribution to the international literature with respect to the following aims.

### Aims of Study

(1). To identify changes in the social and emotional difficulties reported by the primary caregivers of children aged 5 and again at 9 years of age and also those reported by the child’s teacher.
(2). To contrast differences in child and parental characteristics for children who have persistent social-emotional difficulties or who had been reported to have them at either age.
(3). To assess the variables that most differentiate children with social-emotional difficulties from those who had had no reported difficulties.

The study is based on secondary analysis of publicly available, anonymised children and family data that were obtained as part of a longitudinal study: called Growing Up In Ireland. Full details of the study and the measures used is available at: https://www.growingup.ie/ (accessed on 21 July 2021).

## 2. Materials and Methods

### 2.1. Country Context

Ireland had a population of 4.59 million, with 66,700 children aged 5 in 2013 and 71,300 aged nine in 2018. Children generally commence free primary education before age 5. Pupils with additional needs can be referred by parents and schools for assessment and therapeutic services provided by educational psychologists and community health services. Children with special education needs may receive additional support within mainstream schools or in special units and schools. Ireland is classed by the World Bank as a High Income country.

### 2.2. Growing Up in Ireland (GUI) Longitudinal Study

The longitudinal study commenced in 2008/09 when the participating children were nine months of age and subsequently followed when they were three, five and nine years of age [14]. At nine months of age a total of 11,134 children were recruited to the GUI study representing a response rate of 65% of all families approached and 69% of valid contacts were made in the course of the fieldwork. The families were chosen randomly from the list of the 41,185 infants registered on the Child Benefit Register—a universal benefit payable to each child in an Irish family—born between 1st December 2007 and 30th June 2008.

The obtained sample was weighted at each data collection point to ensure that the structure of the completed sample was in line with the original population from which it has been selected. A total of 11 main characteristics of the infant and his/her family were used in the generation of the weights [14]. As is common with longitudinal studies, sample attrition occurred so that by age 5 there were 9793 children (81% of the original sample) and at 9 years there were 8302 participants (72% of the starting sample). However for the purposes of this study, information was available for 7676 children and families whose data on social emotional difficulties was complete at ages 5 and 9 years. This sample too was weighted to ensure it matched the original population. The merger of information obtained at ages 5 and 9 was undertaken using guidance provided by the GIU study.

### 2.3. Information Gathered

The initial contact with the family was made via a letter and information sheet from the GUI Study Team. The interviewer subsequently made a personal visit to each household to arrange an interview. At that first visit, interviewers asked to speak to the study child’s primary caregiver (PC) (usually the mother, but for example, fathers and grandmothers may also take this role). After answering any queries the PC had, the interviewer asked them to sign two copies of the main consent form; one of which the PC retained. Only after securing signed consent did the interviewer commence the information gathering. This included an interview with the child’s primary caregiver (using computer assisted personal interviewing via a lap-top) and self-completion questionnaires with the PC (using computer-assisted self-completion software on a lap-top). In addition, self-completion questionnaires were sent, with parental permission to the child’s teacher to obtain further information about the child. These were completed in writing and posted back to the Study Team.

The questions were developed in consultation with various international experts and local personnel and revised in the light of their use in earlier waves of data gathering. Pilot testing was also undertaken of the measures prior to their use with each age group. Although the authors had no control over the information gathered in the GUI study, fortuitously the study had included a reputable indicator of children’s social-emotional difficulties from age four onwards as described below.

The remainder of this section describes the information pertinent to this study, obtained in 2013/14 at age 5 and in 2017/18 when children were aged 9.

#### Strengths & Difficulties Questionnaire (SDQ)

The SDQ is a widely used, brief (25 item) screening questionnaire designed to assess emotional health and problem behaviours in children It has reasonable reliabilities and validity especially for the total scores [13,15,16]. A total score is calculated based on four of the five subscales with a threshold score of 17 and above (out of a possible 40) as ‘abnormal’ and requiring further investigation [12]. The scale was completed by the PC when the child was aged 5 and again at 9, and also by the child’s teacher at each age level. On each assessment, the children were grouped into those above and below the threshold score. The Cronbach Alpha for the total scale score in the sample used in the present study for children at age 9 was 0.74 for parents and 0.87 with teachers [12].

### 2.4. Possible Associated Variables

In line with the bio-psycho-social model of children’s development, various additional pieces of information were available from the GUI dataset [17,18]. These can be grouped into three domains.

#### 2.4.1. Demographic Information about the Family

This was obtained when the child was 5 years old and updated at age 9; including details of lone parenting, the educational level of the primary caregivers, the socio-economic background of the family and the equivalized income level when all sources of income are counted including social security benefits. The child’s gender was recorded.

#### 2.4.2. Parental/Teacher Relationships and Parental Wellbeing

The following measures were completed:

##### The Pianta Scales—Child Parent Relationship Scale (CPRS) and Student-Teacher Relationship Scale (STRS)

Each of these 15-item scales assess both the positive and negative aspects of the relationship between either parent and child or teacher and child by using the same items for each [19]. The scales were completed by the PC at age 5 and again at age 9 and by the child’s teacher at the same ages. For the purpose of this analysis, only the conflict scale (range 8 to 40) was used. A higher score is indicative of greater conflict. The Cronbach alpha for the primary caregivers of 9-year old children was 0.79. The alpha for teachers of 9-year old children was: 0.86 [12].

##### Parental Stress Scale

A six-item Parental Stressors sub-scale from the above test was used with primary caregivers using five point ratings [20]. A total score (range 6–30) was calculated with high scores indicative of greater stress. The Cronbach alpha for this subscale at Wave 5 was 0.78 [12].

##### Centre for Epidemiological Studies Depression Scale (CES-D)

These eight questions provide a short, self-report screening instrument for depression in the general population [21]. Primary caregivers answered these items only as part of the self-complete questionnaires when the child was aged 9. Scores ranged from 0 to 8 with higher scores indicative of depression. The Cronbach alpha at Wave 5 was 0.85 in this sample [12].

#### 2.4.3. Children’s Developmental Impairments

Teachers noted from a prepared list the impairments that “limited the child’s activity at school”. The numbers and percentages of children reported for each condition based on weighted data is also given. The impairments recorded on the GUI dataset were: Physical disability or visual or hearing impairment (*n* = 200:3.0%); Speech impairment (*n* = 200:3%); Autism spectrum disorders (*n* = 228:3.4%); General learning disability (mild) (*n* = 348:5.2%); General learning disability (moderate/severe/profound) (*n* = 113:1.7%); Specific learning difficulties (e.g., dyslexia) (458:6.8%).

Based on the frequencies of children reported as having each impairment, three groupings were created: children with no developmental impairments (*n* = 5889:84.2%); those with one impairment reported (*n* = 824, 11.8%) and those with two or more impairments reported (range 2 to 6) (280:4.0%).

In addition, the teachers also identified children with emotional or behavioural problem (e.g., Attention Deficit [Hyperactivity] Disorder—ADD, ADHD) (*n* = 273:4.2%). Following Swift et al. [12], emotional behavioural problems were omitted from the above categorization of developmental impairments as it might confound the ratings of SDQ which is the main dependent variable.

Teachers did not return information on 659 (8.8%) of children whose primary caregivers have provided information at both ages 5 and 9.

### 2.5. Data Analysis

The information obtained when the children were aged 5 was merged with that obtained at age 9 on a SPSS database. Weightings were applied in accord with the algorithm developed for the Growing Up in Ireland study. This means that the number of cases reported throughout this paper are slightly different from the information gathered at each age level.

Using SPSS (version 25), frequency counts and bivariate analyses were undertaken including Chi Sq tests and ANOVAs as appropriate. Effect sizes were also estimated. Multinominal logistic regression was used to identify the predictors of children with above the threshold scores on SDQ at both ages and at one of these ages based on primary caregiver ratings and replicated with teachers’ ratings.

## 3. Results

Table 1 presents the number and percentages of children reported by primary caregivers grouped by SDQ total scores when aged 5 and at age 9.

As the table shows, the bulk of children had low scores at both ages. However, 5.4% of children scored above the threshold at age 9 although they had not done so at age 5. Conversely 3.1% of children no longer scored above the threshold at age 9 although they had done so at age 5. This left 2.9% who scored above the threshold at both ages.

Three groupings were formed to facilitate further statistical analysis: those children who were below the threshold at both time points (*n* = 6802:88.6%); those who had been above the threshold at either age 5 or age 9 (*n* = 655:8.5%) and those who scored above the threshold at both ages (*n* = 219:2.9%).

### 3.1. Predictors of Change

Table 2 summarizes the relationships between the three groups and a number of relevant predictor variables that were available in the GUI dataset. As well as the statistical significance of the differences, the effect sizes are also reported using the common conventions. The shaded areas of the table indicate groupings of variables related to child characteristics, primary caregiver and family demographics, and measures relating to primary caregivers when children were aged 9 and also at age 5. Teacher ratings on conflict are also given.

In terms of children’s characteristics the largest effect size on SDQ groupings was for children whom teachers had rated as having emotional and behavioral problems such as ADHD. Even so, only a minority of these children (30.7%) scored above the threshold at both ages. Nonetheless the pattern of responses for children having these problems with teacher ratings on the SDQ does help to validate the SDQ threshold as identifying children worthy of further investigation.

Higher proportions of children with one or more other developmental impairments also had above threshold scores at both ages. However, the child’s gender had a smaller effect; with boys more likely than girls to be above the threshold at both ages.

In terms of primary caregiver and family characteristics, the effect sizes were generally small, with above threshold scores for children more likely to be reported in lower income families, those in unskilled jobs or unemployed, for primary caregivers with lower levels of education and for single parents.

Primary caregiver’s ratings of conflicts with the child were higher for children with above threshold scores at both age 9 and age 5. Stress scores of parents were higher with children who had above threshold scores at both ages. At age 9, primary caregivers reported higher levels of depression (this information was not asked at age 5).

### 3.2. Logistic Regression Analysis

In order to control for the inter-relationships among the above variables, a multinominal logistic regression was used to assess the contribution of selected variables to differentiating children who had scores above the 17+ threshold at either age 5 or age 9 and those who had scored above at both ages. In both instances the comparison was with children who had scored below the threshold at each age. All independent variables in the model were measured at 9 years (Wave 5). Household class was omitted from the final multivariate model on account of the high level of empty cells it caused, with equivalized household income deemed an acceptable alternative. To address positive skew, a square root transformation was performed on the parental depression variable. Finally, outliers in the conflict variable were addressed by winsorizing scores at 2.5 standard deviations.

Due to missing data on one or more of the remaining variables, the sample was reduced to 6277 (unweighted) cases with 1423 missing cases (18.5%). The bulk of missing cases came from missing data on family income (*n* = 587:7.6%) and missing teacher’s information on developmental impairments (*n* = 588:7.6%). Checks were then undertaken to determine if there were any significant biases in these missing cases on the variables included in the regression. There were significant differences between both missing income and missing impairment data and whether there was a partner in the home, between income and the primary caregiver’s level of education, and between missing teacher responses on developmental impairment and parental stress (all measured at 9 years). However, there were no significant differences between the missing data and the dependent variable, and on this basis these variables were retained in the final model.

The final model was a significant fit for the data (Chi sq 1412.35: df 18: *p* < 0.001 and accounted for 35.6% of the variance (Nagelkerke R^2^). Table 3 details the significance of the predictors and the odds ratios.

The regression analysis identified a number of variables that were significantly associated with children who scored above the threshold at ages 5 and/or age 9. Children with one or more developmental impairments were up to 2.6 times more likely than those without developmental impairments to score above the threshold at either 5 or 9 years. These odds increased greatly for children who scored above the threshold at both 5 and 9 years when the odds were 5.7 times those for children without developmental disabilities. Furthermore, males were no more likely than females to be above the threshold at either 5 or 9 years but males had a significantly increased odds ratio for being above the threshold at both ages.

The lower educational attainment of primary caregivers—those who had not attended post-secondary education or university—compared to those with university degrees, increased the odds ratios for children being above the threshold either at 5 and/or 9 years and especially at both ages. Lone parenting was also a significant predictor but only for children who scored above the threshold at age 5 or 9. Likewise low income was a significant predictor for those scoring above the threshold at 5 and 9 but not for children who scored above the threshold at one of these time points.

Some of the parent ratings also contributed significantly to the regression model; notably ratings of conflict with the child at age 9 for both groups of children who had above threshold scores. Ratings of parental stress and depression were also significant at age 9.

These relationships broadly replicate those reported by Swift et al. [13] for changes in SDQ thresholds for children aged 9 to 13.

### 3.3. Replication with Teachers

The findings based on the SDQ scores provided by children’s primary caregivers can be replicated to an extent with similar ratings provided by teachers who also completed SDQ ratings at ages 5 and 9 along with ratings of conflict in their relationships with the child. However, it should be noted that the children mostly likely would have had different teachers at the two ages whereas almost all primary caregivers were constant.

Based on teacher’s ratings, three groupings were formed: those children who were below the threshold at both time points (*n* = 5944:88.6%); those who had been above the threshold at either age 5 or age 9 (*n* = 628:9.4%) and those who scored above the threshold at both ages (*n* = 135:2.0%).

A logistic regression was performed with the same independent variables as for the earlier model and, as before, missing data on several of the independent variables reduced the available cases for analysis to 5917. On this occasion, missing data on the developmental impairment variable was significantly associated with the dependent variable, with children scoring below the threshold on the SDQ at age 5 and 9 years significantly less likely to be missing than their peers who had above threshold SDQ scores at either age 5 or age 9. Due to the goal of comparison, and the importance of this variable to the analysis, it has been retained. However, this potential source of bias should be born in mind.

This second model was also a significant fit for the data (*n* = 5557: Chi sq 1103.61: df 18: *p* < 0.001) and in terms of Nagelkerke R^2^ it accounted for 33.8% of the variance. Table 3 details the significance of the predictors and the odds ratios.

A similar pattern of odds ratios as reported for the primary caregivers was also present for teachers in terms of children with one or more developmental disabilities having greater odds of being above the threshold at either age 5 or 9 and more especially at both ages. The teacher’s perspective on whether or not a child’s SDQ is above the threshold at age 5 or 9 years is also significantly associated with gender, with boys being more likely as girls to score above the threshold at either age.

Replacing primary caregiver with teacher SDQ responses as the dependent variable reduces the significance of the parental stress and depression scales in the model although other family characteristics did contribute significantly to the regression model. However, conflict in the teacher-child relationship continues to play a significant role in predicting above-threshold scores at either or both waves.

## 4. Discussion

This study had four main strengths. It was based on a large, representative sample of Irish children aged five and nine years along with their primary caregivers. The children and families were followed over a five-year period to examine changes in social-emotional difficulties. The variables associated with the greater likelihood of children having social-emotional difficulties were identified. Reports of the children’s difficulties were sourced from both primary caregivers and their school teachers to provide a replication of the findings.

The study found that a small proportion of children—around 1 in 20—at age five showed difficulties above the threshold on the SDQ that warranted further investigation. This confirms the results from studies in other countries [1] although these investigations also provide evidence of these difficulties occurring with preschool children [7,10]. Although the proportion of children with marked social-emotional difficulties may be small, when it is applied to the population of 5-year olds in Ireland, around 4000 children could be predicted to have above threshold scores.

However, for around half of these children, the difficulties were no longer considered of concern when the children were aged nine years. Two caveats need to be made to this encouraging finding. Because of the time interval, there may be fluctuations in the difficulties they present across the five-year period. Also, no information was available as to whether the children or families had received any interventions aimed at ameliorating their behaviours. These have been shown to have some effect; a point we will return to later [22].

By contrast though, there were children who scored above the threshold at age 9 but had not done so when aged 5 years. Indeed, the proportion of nine-year olds rated above the threshold had risen to 1 in 12 (8%) children. This equates to around 6000 nine-years old nationally and suggests that around 25,000 Irish children between the ages of 5 and 9 years would have above threshold ratings on the SDQ. The increasing proportions of children with higher ratings of social-emotional difficulties with increasing age has been reported in other studies [4]. However, an earlier cohort of nine-year old children in Ireland who were followed up at 13 years [11] found that 7% of nine year olds had above threshold scores which dropped slightly to 6.3% of 13 year olds.

Of particular concern are the children who continued to have above threshold scores at both ages—2.9% of the sample—and over 2000 nationally. This is exactly the same percentage of children who were reported to have maintained above threshold scores in an earlier cohort of Irish children from ages 9 to 13 years [13]. As noted above, it could be that the children’s ratings may have fluctuated between the ages of 5 through to 13 but even so this data would suggest that any interim improvement does not appear to be stable.

A further outcome of the present study is the identification of the children who are most likely to have continuing social-emotional difficulties. Moreover, these analyses were broadly replicated for ratings given by primary caregivers and by the child’s teacher. The foremost association with social-emotional difficulties was the child having one or more developmental impairments. These children were up to six times more likely to have above threshold scores at both ages based on primary caregivers’ ratings and over seven times more likely on the teachers’ ratings. Boys rather than girls also had higher odds, ratios especially on teacher ratings, but these odds were much lower than for impairments.

Family factors were also associated with the children’s social-emotional difficulties; notably the children whose primary caregivers had lower levels of education were four times more likely to have continuing difficulties based on primary carer ratings and twice as likely based on teacher ratings. Families with lower incomes were also twice as likely to have above threshold ratings made by primary caregivers and teachers. Lone parenting was associated with higher scores in teacher ratings but not in those given by primary caregivers.

In addition, there was some evidence of an association between the children’s difficulties and the wellbeing of primary caregivers; notably in terms of conflict in their relationship with the child, with higher stress and depression scores. However on all these measures the odd ratios were small, albeit they were statistically significant. Teachers also reported a significantly higher odds ratio for conflict in their relationship with the child.

This pattern of odds ratio was also present for children who had above threshold scores at either age 5 or age 9 but the odds ratios were lower as shown in the lower part of Table 3. This is further confirmation of the significant influences on children’s social and emotional development. Moreover the pattern of odds ratio associated with SDQ thresholds replicates those reported previously for another cohort of Irish children aged 9 and 13 years with respect to children’s impairments, lower education of primary caregivers and higher scores on conflicted relationship but not for child’s gender which was not a significant contributor to the models in this cohort [13]. Past studies in other countries and with similar aged samples have also noted the strong association between children’s developmental impairments and social-emotional difficulties [4].

Unfortunately, no information was available of the services and supports made available to these children, their families and teachers. It is likely that some children may be prescribed medications although their long-term use is not encouraged nor is their administration to young children recommended [23]. However, there is growing evidence that behavioral interventions involving a multi-disciplinary team of professionals can be effective in managing the children’s behaviour in school and at home [8]. Also supports need to be provided to family caregivers to help build their coping strategies and enhance their emotional well-being. In Ireland, as in many other countries, such family-centred resources are not widely available and rarely on a preventative basis at a younger age [24]. Rather health and social services are focused on managing adolescents in crisis when higher levels of support are needed over longer periods with consequently greater costs. There are also increased risks of self-harming and suicide with older children and teenagers with emotional difficulties as well as anti-social behaviours and offending that leads to involvement with the criminal justice system [25,26].

Descriptive studies using existing datasets have several limitations. The information gathered was not specifically designed to capture data around children’s social-emotional difficulties, largely because such children form only a minority of the child population. In such circumstances, the recruitment of booster samples of children with additional needs would also help to increase the statistical power of the comparative analyses undertaken and would help to reduce the range within the odds ratio reported in this and other studies [27]. In addition, more detailed assessment tools would help to identify diverse patterns of socio-emotional difficulties that may require different intervention strategies [28].

In terms of clinical implications, there would be merit in screening five-year olds for social-emotional difficulties using the SDQ or a similar scale [12,29] and especially when a developmental impairment has been identified. Moreover, ratings from primary caregivers and teachers would help to identify behaviours that occur in the home as well as school. Low level interventions such as online courses or short training workshops could be offered to families; organized by schools and/or by health personnel such as psychologists and therapists [30]. More intensive interventions could be reserved to children and families whose personal circumstances make it difficult to avail of group-based supports, or those who require individualized and more intensive support. Similar opportunities could also be offered to older children throughout their schooling but better to start young before children’s behaviours and the family dynamics become established [31].

Further research is needed to refine the screening process which could be adopted on entry to preschool and schools along with an evaluation of various levels of intervention provided to children, primary caregivers and teachers. A cost-efficiency analysis should be undertaken. Future longitudinal studies should attempt to evaluate the longer term impact of early interventions on children’s social-emotional difficulties and their engagement with education, health and social services and juvenile justice services [32].

## 5. Conclusions

Although the number of Irish children presenting with continuing social-emotional difficulties from ages 5 through 9 years is small, they can present an ongoing societal cost in terms of their future development, the impact on family relations and demands placed on educational, health and social services. This study identified the children and families who are at greatest risk and for whom targeted early interventions could be provided at a relatively low cost [33].

## Figures and Tables

**Table 1:** The number and percentage of children in the subgroupings based on SDQ scores.

	Age 9 Groupings		
Age 5 Groupings	Below 17	17+	Total
Below 17	6802	417	7219
	88.6%	5.4%	94%
17+	238	219	457
	3.1%	2.9%	6.0%
Totals	7040	636	7676
	91.7	8.3%	100.0%

**Table 2:** The percentage of children or mean scores in each of the three subgroups for the predictor variables listed.

Predictor Variables		Below Threshold 17+ (*n* = 5879)	Either above 17+ at Age 5 or 9 (*n* = 824)	Above 17+ Age 5 and Age 9 (*n* = 279)	Statistical Tests
Gender	Male Female	50.2% 49.8%	57.8% 42.2%	68.9% 31.1%	Chi Sq 41.74, df 2, *p* < 0.001 Cramer’s V 0.074 +
Developmental Impairments ~	None One +	87.1% 12.9%	66.7% 33.3%	46.8% 53.2%	Chi Sq 385.4, df 2, *p* < 0.001 Cramer’s V 0.235 ^
Developmental Impairments ~	None One 2 +	87.1% 10.2% 2.7%	66.7% 21.7% 11.6%	46.8% 32.0% 21.2%	Chi Sq 450.70, df 4, *p* < 0.001 Cramer’s V 0.180 +
Emotional/Behavioural problems ~	Yes No	1.9% 98.1%	18.2% 81.8%	30.7% 69.3%	Chi Sq 712.67, df 2, *p* < 0.001 Cramer’s V 0.326 *
Partner in the home	No Yes	13.1% 86.9%	26.7% 73.3%	24.7% 75.3%	Chi Sq 106.29, df 2, *p* < 0.001 Cramer’s V 0.118 +
Social Class	Profes./Manag. Other non manual/ skilled manual	56.0% 33.2%	42.5% 41.7%	27.7% 47.2%	Chi Sq 92.22, df 2, *p* < 0.001
	Semi unskilled/manual	10.9%	15.7%	25.2%	Cramer’s V 0.081 +
PC Education	Secondary Post-secondary University	25.9% 41.3% 32.8%	36.0% 42.4% 21.6%	48.8% 39.6% 11.5%	Chi Sq 111.81, df 4, *p* < 0.001 Cramer’s V 0.085 +
Income	Lowest 30% Highest 70%	27.8% 72.2%	41.6% 58.4%	53.5% 46.5%	Chi Sq 106.72, df 2, *p* < 0.001 Cramer’s V 0.124 +
PC rating—Conflict Age 9		14.17 (SD 5.30)	20.70 (SD 6.87)	26.09 (SD 6.46)	Welch F = 621.04 (2, 461.45) *p* < 0.001 (Eta Squared 0.186 +)
PC Stress score Age 9		13.00 (SD 4.27)	15.98 (SD 4.74)	17.60 (SD 4.79)	Welch F = 207.13 (df 2, 462.83) *p* < 0.001 (Eta Squared 0.061 ^)
PC Depression Score Age 9		2.085(SD 2.89)	3.94 (SD 3.81)	5.45 (SD 4.49)	Welch F = 128.84 (2450.28) *p* < 0.001 (Eta Squared 0.06 ^)
PC rating—Conflict Age 5		14.33 (SD 5.26)	18.82 (SD 6.41)	25.31 (SD 5.81)	Welch F = 513.51 (2, 466.45) *p* < 0.001 (Eta Squared 0.139 +)
PC Stress score Age 5		11.38 (SD 3.84)	14.11 (SD 4.58)	17.40 (SD 5.19)	Welch F = 241.78 (2, 453.06) *p* < 0.001 (Eta Squared 0.088 ^)
Teacher ratings—Conflict Age 5		10.53 (SD 3.52)	16.48 (SD 7.43)	21.07 (SD 7.92)	Welch F = 276.73 (2, 271.92) *p* < 0.001 (Eta Squared 0.219 ^)
Teacher ratings—Conflict Age 9		11.01 (SD 3.87)	17.76 (SD 7.84)	23.74 (SD 8.00)	Welch F = 341.07 (2, 272.55) *p* < 0.001 (Eta Squared 0.243 ^)

~ this information was not provided by teachers for 691 children, hence the reduced numbers. + small effect ^ medium effect * large effect.

**Table 3:** Summary table for the Regression analysis on SDQ ratings by Primary Caregivers (PC) and by teachers.

	Primary Caregivers Ratings				Teacher Ratings			
	Model B (SE)	Lwr	Odds Ratio	Uppr	Model B (SE)	Lwr	Odds Ratio	Uppr
**Above the Threshold at age 5 and age 9 years**								
Disability *(ref: no impairment*)								
1+ impairments	1.747 (0.182) ***	4.015	5.737	8.199	1.865 (0.219) ***	4.201	6.459	9.929
Gender *(ref: girls)*								
Boys	0.55 (0.186) **	1.203	1.733	2.495	0.082 (0.235)	0.685	1.085	1.720
Primary caregiver education *(ref: University degree)*								
Secondary or below	1.386 (0.286) ***	2.285	4.000	7.002	0.853 (0.322) **	1.249	2.346	4.408
Post Secondary	0.845 (0.275) **	1.357	2.328	3.995	0.286 (0.315)	0.717	1.330	2.468
Partner in the home *(ref: Yes)*								
No	0.081 (0.222)	0.702	1.084	1.674	0.401 (0.245)	0.924	1.493	2.411
Equivalised household income *(ref: highest 70%)*								
Lowest 30%	0.689 (0.194) ***	1.361	1.992	2.915	0.387 (0.248)	0.905	1.473	2.397
**Rating Scales**								
Pianta Child-Parent -conflict (Model 1) Pupil-teacher conflict (Model 2)	0.284 (0.017) ***	1.283	1.328	1.374	0.339 (0.023) ***	1.342	1.404	1.468
Parental Stress Scale	0.065 (0.021) **	1.025	1.067	1.112	0.039 (0.025)	0.991	1.039	1.091
Depression Scale (transformed)	0.425 (0.085) ***	1.296	1.529	1.805	0.024 (0.100)	0.843	1.025	1.245
**Above the threshold at *either* age 5 *or* age 9 years**								
Disability *(ref: no impairment)*								
1+ impairments	0.968 (0.112) ***	2.114	2.632	3.277	0.981 (0.119) ***	2.112	2.666	3.367
Gender *(ref: girls)*								
Boys	0.189 (0.101)	0.992	1.208	1.471	0.497 (0.117) ***	1.308	1.644	2.067
Primary caregiver education *(ref: university degree)*								
Secondary or below	0.557 (0.144) ***	1.315	1.745	2.317	0.494 (0.153) ***	1.214	1.640	2.214
Post Secondary	0.420 (0.128) **	1.184	1.521	1.956	0.216 (0.140)	0.943	1.241	1.634
Partner in the home *(ref: Yes)*								
No	0.639 (0.123) ***	1.488	1.895	2.413	0.336 (0.137) *	1.068	1.399	1.831
Equivalised household income *(ref: highest 70%)*								
Lowest 30%	0.200 (0.114)	0.977	1.222	1.528	0.252 (0.124) *	1.008	1.286	1.642
**Rating Scales**								
Pianta Child-Parent -conflict (Model 1) Pupil-teacher conflict (Model 2)	0.142 (0.009) ***	1.133	1.152	1.172	0.118 (0.053) ***	1.184	1.207	1.230
Parental Stress Scale	0.057 (0.012) ***	1.033	1.058	1.084	0.042 (0.013) ***	1.017	1.043	1.069
Depression Scale (transformed)	0.249 (0.048) ***	1.168	1.283	1.410	0.018 (0.053)	0.917	1.018	1.131
**Model Fit**								
Model X^2^	X^2^ (18) = 1412.35 ***				X^2^ (18) = 1103.61 ***			
Nagelkerke R^2^	0.356				0.338			

*** *p* < 0.001 ** *p* < 0.01 * *p* < 0.05.

## Data Availability

The data used in this study is available in request from the Irish Social Sciences Database: https://www.ucd.ie/issda/ (accessed on 21 July 2021).
